# Groundwater chemistry affected by trace elements (As, Mo, Ni, U and V) from a burning alum shale waste deposit, Kvarntorp, Sweden

**DOI:** 10.1007/s11356-021-12784-2

**Published:** 2021-02-14

**Authors:** Kristina Åhlgren, Viktor Sjöberg, Bert Allard, Mattias Bäckström

**Affiliations:** Man Technology Environment Research Centre, Örebro University, SE-701 82 Örebro, Sweden

**Keywords:** Black shale, Groundwater chemistry, Trace elements

## Abstract

Worldwide, black shales and shale waste are known to be a potential source of metals to the environment. This project demonstrates ongoing weathering and evaluates leaching processes at a 100-m-high shale waste deposit closed in the 1960s. Some deep parts of the deposit are still burning with temperatures exceeding 500 °C. To demonstrate ongoing weathering and leaching, analyses of groundwater and solid samples of shale and shale waste have been undertaken. Largest impact on groundwater quality was observed downstream the deposit, where elevated temperatures also indicate a direct impact from the burning waste deposit. Groundwater quality is largely controlled by pH and redox conditions (e.g., for arsenic, nickel, molybdenum, uranium and vanadium), and the mixture of different waste materials, including pyrite (acidic leachates) and carbonates (neutralizing and buffering pH). Analyses of shale waste from the deposit confirm the expected pyrite weathering with high concentrations of iron, nickel and uranium in the leachates. No general time trends could be distinguished for the groundwater quality from the monitoring in 2004–2019. This study has shown that black shale waste deposits can have a complex long-term impact on the surrounding environment.

## Introduction

Black shale is the denomination of a heterogeneous group of dark-coloured sedimentary rocks containing organic matter (Vine and Tourtelot 1969) which to various extents are enriched in sulfides and metals. Ketris and Yudovich (2009) have made estimates of median concentrations based on reported data in black shales globally, e.g., 30 mg/kg for arsenic, 20 mg/kg for molybdenum, 70 mg/kg for nickel, 8.5 mg/kg for uranium and 205 mg/kg for vanadium. Weathering and leaching of shale residues on mining sites and from shale waste dumps lead to release of elements resulting in local and regional environmental impacts. This is reported from numerous sites worldwide, e.g., in Denmark, Norway, Finland, Estonia and Germany in Europe and also from the USA and China (e.g., Loukola-Ruskeeniemi et al. 1998; Puura 1998; Puura et al. 1999; Woo et al. 2002; Peng et al. 2004; Burkhardt et al. 2009; Grawunder et al. 2009; Jüriado et al. 2012; Paikaray 2012; Peng et al. 2014; Schovsbo et al. 2014; Phan et al. 2015; Liu et al. 2017; Lerat et al. 2018; Parviainen and Loukola-Ruskeeniemi 2019; Stuckman et al. 2020; Waersted et al. 2020).

The black shales in Sweden (denoted alum shales) are mainly composed of muscovite-illite, quartz and feldspar with up to 15% pyrite and up to 20% organic material (kerogen). The shales are enriched in trace elements such as molybdenum, nickel, uranium and vanadium (Armands 1972; Andersson et al. 1985). Mining of alum shale from the 18th to the 20th centuries has led to dispersion of elements from weathering of shale residues, still in progress, notably at Degerhamn, Ranstad and Kvarntorp (e.g., Allard et al. 1991, 2011; Falk et al. 2006; Kalinowski et al. 2006; Lavergren 2008; Lavergren et al. 2009; Åhlgren et al. 2020).

In Kvarntorp, 200 km south-west of Stockholm, Sweden, alum shale was mined in open pits and processed for oil production during 1942–1966. Most of the shale residues from the mining period (unprocessed fine-grained shale denoted fines, semi-combusted shale denoted semi-coke and fully burned shale denoted shale ash, as well as minor amounts of limestone) were deposited on site, either backfilling of open pits, or on a single deposit, denoted Kvarntorpshögen (the Kvarntorp waste deposit). The deposit is presently 100 m high and covers an area of 450,000 m^2^. It is estimated that the deposit contains 3 Mtonnes of fines, 2 Mtonnes of semi-coke and 23 Mtonnes of shale ash, as well as limestone in some parts (Bäckström 2010). The deposit was closed in 1966, but temperatures are still elevated, caused by pyrite oxidation in the fines (temperatures up to 100 °C), as well as kerogen oxidation (temperatures above 500 °C).

It is of general interest to monitor the release of leachates from shale residues, particularly from well-defined deposits, for several reasons: (1) assessment of present and future environmental impact and health risks associated with spreading of elements from the deposit and (2) enhanced understanding of relevant processes—weathering and leaching in situ, element mobilization and transport, element accumulation in soil and sediments.

The Kvarntorp site is of interest for extended studies of metal releases from alum shale in situ for several reasons: (1) the alum shale in Kvarntorp generally contains higher trace element concentrations than the global median levels except for arsenic which is quite similar and nickel with large variations in concentration (cf. Allard et al. 2011). (2) There is a continuously progressing weathering of shale in the deposit. Oxidation of pyrite and other metal sulfides leads to releases of metals by run-off and into the local groundwater aquifers. This has been going on for more than 50 years. (3) The deposit is still burning which means that large parts of the deposit are dry. Increased leaching of metals from the area is expected after cooling of the deposit and water exposure to parts that presently are dry. (4) It may be possible to assess the reactivity and leachability in situ of different shale fractions—fines, semi-coke and shale ash. (5) No remediation activities have been undertaken to control and reduce metal releases from the deposit since the end of mining 54 years ago. Element immobilization due to sorption and secondary mineral formations are important factors that can be demonstrated, and internal drainage mixing processes can be assessed (cf. Vriens et al. 2019).

The objective of the present project is to demonstrate the ongoing weathering and leaching processes in the Kvarntorp alum shale waste deposit and the resulting impact on the local and regional groundwater quality. Present and long-term impacts on groundwater chemistry are assessed largely based on groundwater quality monitoring in the vicinity of the deposit starting in 2004.

## Description of the area

**Fig. 1:**
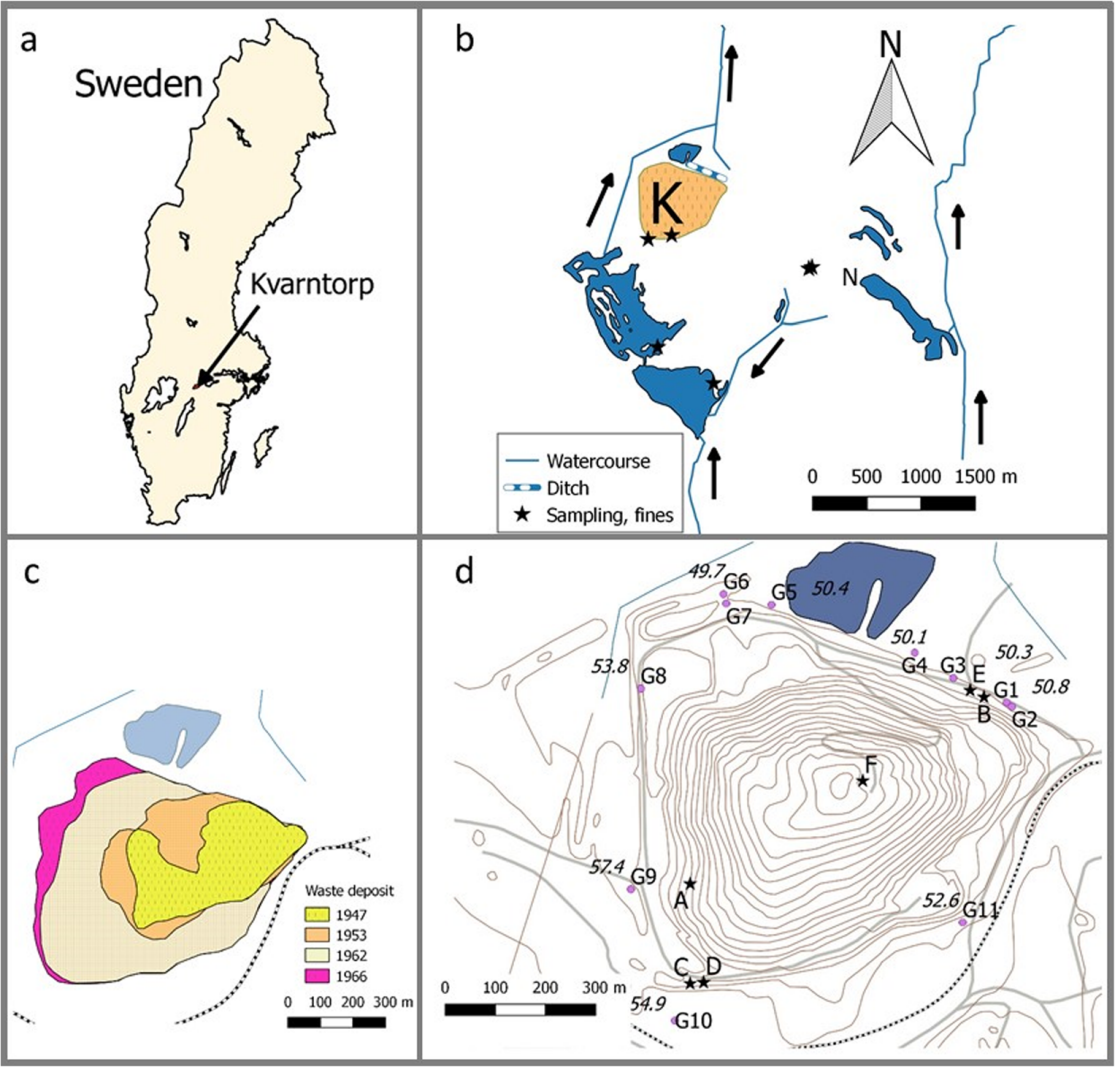
**a** Map showing the location of Kvarntorp. **b** Map showing the Kvarntorp area with the waste deposit Kvarntorpshögen (K) and the pit lake Norrtorpssjön (N), location for sampling of shale and limestone. **c** Extent of the waste deposit from 1947 to 1966, when operations ended. Final size: 100 m high, covers an area of 450,000 m^2^, contains some 3 Mtonnes of fines, 2 Mtonnes of semi-coke and 23 Mtonnes of shale ash. **d** Location of groundwater wells G1–G11 around the deposit and locations for sampling of solid samples (A, B = shale ash; C, D = fines (D used in humidity cells, Sartz et al. in prep), E = shale ash (red ash used in humidity cells) and F = shale ash (black ash used in humidity cells)). Average levels for groundwater depths in the wells (metres above sea level) are given. (Maps are processed with the software QGIS Desktop 2.14.13, with data from Lantmäteriet (Swedish Land Survey) as a basis)

The location of Kvarntorp in Sweden is shown in Fig. 1a, as well as a map showing the mining area with the shale waste deposit (Kvarntorpshögen), pit lakes and watercourses leading in to and out from the area (Fig. 1b).

The crystalline basement in the area is superimposed by lower Cambrian Mickwitzia sandstone followed by Lingulid sandstone beneath layers of phosphorite and glauconite sandstone. Higher up in the strata, green shale (mudstone) is followed by late Cambrian alum shale and Ordovician limestone covered by till on top (Bengtson 1971; Nielsen and Schovsbo 2007, 2015). The uppermost and youngest layers have been eroded and are not represented all the way to the northern limit of the sedimentary outlier since the bedrock is tilted towards the south (Hessland and Armands 1978).

A marshy meadow in a small valley in the northern part of the area contains no alum shale. The top layer of the bedrock is a 5- to 6-m-thick layer of the green shale above the sandstone layers, covered by 4–5 m of till. This area became the location for the shale waste deposit (Kvarntorpshögen) in 1942. The green shale constitutes an almost impermeable layer under the deposit that largely prevents water exchange between the deposit and the sandstone aquifer (Holm et al. 2005). Fig. 1c shows the evolution of the deposit during 1947–1966. The exact locations in the deposit and the quantities of the various shale residues (fines, semi-coke and shale ash) are not known since the records about the waste are sparse or non-existing.

The locations of groundwater wells G1–G11 around the deposit are given in Fig. 1d. These wells were installed in 1997 (G11), 2003 (G4 and G5) and 2004 (G1–G3 and G6–G10).

## Material and methods

### Shale sampling

Solid samples, two shale ashes (A and B) and fines (C), were collected from the waste deposit (Åhlgren et al. 2018) (see Fig. 1d for locations) and from an outcrop at an open pit (five samples of alum shale and two samples of limestone; location N in Fig. 1b). An additional six samples of fines from the deposit were also analysed (see Fig. 1d for location) as well as three alum shale samples from old exploration drill cores kept by the Swedish Geological Survey.

### Groundwater sampling

Two aquifers make up the groundwater in the area—a shallow aquifer in the till underneath and around the deposit with a dominant flow direction from south to north, as indicated by the water levels (Fig. 1d), and a lower aquifer in the sandstone layer (Bengtson 1971). Most of the groundwater wells (G1, G3–G6 and G8–G11) are shallow and installed in the upper aquifer, in the till layer and in the shale waste, while two wells (G2 and G7) are installed in the lower sandstone aquifer (Holm et al. 2005). G2 and G7 were installed using rotational drilling, and the contact between the alum shale aquifer and the sandstone aquifer was sealed with cement. Description of the wells is given in Table 1. Filter sections were generally 1 m long, and washed sand (grain size 0.6–1 mm) was added around the sections. Bentonite was added around the top section in order to prevent infiltration of surface water.

**Table 1:** Description of installed groundwater wells around the waste deposit (Blomquist and Ekholm 1997; Holm et al. 2005)

Well	Depth of well (m)	Pipe	Soil around the filter section	Soil layer above the filter section	Aquifer
G1	6	HDPE	Clay, sand	Shale waste	Upper
G2	31	Steel	Sandstone	Sandstone	Lower
G3	3	HDPE	Shale waste	Shale waste	Upper
G4	3–4.5^a^	HDPE^b^	Shale waste	Shale waste	Upper
G5	3–4.5^a^	HDPE^b^	Sand	Mud, peat	Upper
G6	4.5	HDPE	Till	Shale waste	Upper
G7	31	Steel	Sandstone	Sandstone	Lower
G8	5	HDPE	Oil sludge	Silt^c^	Upper
G9	4.6	Steel	Till	Till	Upper
G10	5	Steel	Till	Till	Upper
G11	6	Plastic^b^	Shale waste	Shale waste	Upper

Upper refers to the upper aquifer in either natural till or shale waste; lower refers to the lower aquifer in the sandstone layers.

^a^Estimated depth

^b^With steel casing

^c^Strong smell of oil

Bailer samplers were used for sampling of water, regularly since December 2015 in all of the wells, G1–G11 (six times annually from 2015 until October 2017 and then three times annually until 2019), totally 176 groundwater samples. Groundwater levels were measured, and the wells were emptied the day prior to sampling. Sterile polypropylene tubes were used for storing the samples at 5–6 °C until analysis. Groundwater temperature was measured in 2017. Additionally, temperature data for G1–G10 from previous studies in 2004 and 2005 were also available (Holm et al. 2005).

Two surface watercourses pass through the area, of which the westerly one is close to the groundwater wells north and north-east of the deposit and may receive run-off water from the deposit (Fig. 1b). A serpentine pond system north of the deposit was constructed in the 1940s, mainly in order to remove organic compounds such as phenols from the industrial process water outlets in the area but is also receiving surface run-off from the deposit. Water quality in the surface waters close to the deposit and in the pit lakes (Fig. 1b) is monitored regularly, as described in Åhlgren et al. (2020) and in Åhlgren and Bäckström (in preparation). The present field program reported here was, however, largely focusing on assessing the direct impact of leachates from the deposit on the local groundwater aquifers, and the impact on surface water quality is outside the present scope.

### Analysis of solid samples

All solid samples were sent to MS Analytical (Vancouver) for total element quantification. Samples were fused with borate flux in a muffle furnace before the resulting beads were dissolved in dilute mineral acid. Inductively Coupled Plasma Optical Emission Spectrometry (ICP-OES) was then used for the analysis of major elements (Al, Ca, Fe, K, Mg, Mn and Na; for detection limits see Table 2). Samples were also digested in aqua regia or in a four-acid mixture and analysed for trace elements (As, Cd, Co, Cu, Mo, Ni, Pb, U and V) by ICP-MS. Total sulfur and carbon were quantified by a Leco carbon and sulfur analyser (for detection limits see Table 2).

**Table 2:** Quantification limits for analysis of solid samples by Leca carbon and sulfur analyser (SPM) and by ICP-MS and ICP-OES (WRA and IMS)

Method	Element	Quantification limit	Unit
SPM-511	C	100	mg/kg
WRA-310	Na_2_O	70	mg/kg
WRA-310	MgO	60	mg/kg
SPM-522	S	100	mg/kg
WRA-310	Al_2_O_3_	50	mg/kg
WRA-310	K_2_O	80	mg/kg
WRA-310	CaO	70	mg/kg
IMS-300	V	10	mg/kg
WRA-310	MnO	80	mg/kg
WRA-310	Fe_2_O_3_	70	mg/kg
IMS-130	Co	0.1	mg/kg
IMS-130	Ni	0.2	mg/kg
IMS-130	Cu	0.2	mg/kg
IMS-130	Zn	1	mg/kg
IMS-130	As	0.1	mg/kg
IMS-300	Sr	0.1	mg/kg
IMS-130	Mo	0.05	mg/kg
IMS-300	Ba	0.5	mg/kg
IMS-300	U	0.05	mg/kg

Quantitative phase analysis by X-ray diffractometry (XRD) was made on the shale samples A, B and C from the waste deposit (Åhlgren et al. 2018). Grinding under ethanol in a vibratory McCrone Micronizing Mill during 10 minutes reduced the sample size to < 10 μm. Step-scan X-ray powder-diffraction data were collected over a range 3–80°2*θ* with CoKα radiation on a Bruker D8 Advance Bragg–Brentano diffractometer equipped with an Fe monochromator foil, 0.6-mm (0.3°) divergence slit, incident- and diffracted-beam Soller slits and a LynxEye-XE detector. The long fine-focus Co X-ray tube was operated at 35 kV and 40 mA, with a take-off angle of 6°. The X-ray diffractograms were analysed using the International Centre for Diffraction Database PDF-4 and Search-Match software by Bruker. The diffraction data were refined with Rietveld program Topas 4.2 (Bruker AXS).

### Analysis of water samples

Electrical conductivity (Radiometer CDC836T-6, with temperature compensation), pH (Metrohm 6.0257.000 with temperature compensation), redox potential (during a few sampling occasions in 2017; Thermo Scientific REDOX/ORP 9678BNWP) and alkalinity were measured in the lab within 12 hours after sampling. Elements were quantified after filtration (0.20-μm polypropylene) and acidification (1 % nitric acid), by Inductively Coupled Plasma Mass Spectrometry (ICP-MS, Agilent 7500cx. Elements and detection limits are given in Table 3. Inorganic anions (chloride and sulfate) were quantified by capillary electrophoresis (2015–2017) using sodium chromate buffer (50 mM) containing TTAB (5 mM) and a 40 cm ∗ 50 μm silica capillary (from 2015 to 2017) or by ion chromatography, SS-EN ISO 10304-1:2009 (2018, 2019).

**Table 3:** Detection limits and used isotopes during ICP-MS analysis of the water samples

Isotope	Element	Detection limit (μg/L)
7	Li	0.013
23	Na	0.792
24	Mg	0.004
27	Al	0.043
39	K	1.734
43	Ca	0.428
51	V	0.001
55	Mn	0.005
56	Fe	0.530
59	Co	0.001
60	Ni	0.010
63	Cu	0.001
66	Zn	0.051
75	As	0.012
88	Sr	0.001
95	Mo	0.001
238	U	0.001

Ferrous iron (Fe(II)) was analysed in 2017 using photometry and the blue complex of Fe(II) and TPTZ (2,4,6-tri(2′-pyridyl)-1,3,5-triazin). A Hewlett Packard 8453 was used, and the absorbance at 595 nm was recorded. To 4 mL of sample was added 0.25-mL HCl (6 M), followed by the addition of 0.5-mL NH_4_F (2 M), 0.5-mL TPTZ (2.4 mM) and finally 1-mL buffer solution (NH_4_Ac (2 M)/HAc (2 M)). The sample was shaken vigorously between each addition. The mix was left for 10 minutes prior to measurement. Calibration was performed by analysis of standards containing Fe(II)(NH_4_)_2_-sulfate, prepared to cover a ferrous iron concentration range of 0.1–11 mg/L.

Chemical speciation calculations were performed on the measured concentrations in all groundwaters, using the geochemical code PHREEQC Interactive (version 3.4.0.12927; Parkhurst and Appelo 1999) with the MINTEQ.v4 database. Saturation indices (SI is the logarithm of the ratio between the ion activity product and the solubility constant) were also obtained from the geochemical calculations.

Principal component analysis (PCA) was performed on all groundwater data, in order to determine the relationship between the different waters, using *The Unscrambler X* (version 10.5) (Camo Software AS, Oslo, Norway). Data from humidity cells (day 7) performed on fines, red ash and black ash from the area (Sartz et al. in prep; sampling locations shown in Fig. 1d) were also included in the PCA in order to determine the impact from different waste materials on the groundwater composition. All data (except pH) was log transformed and normalized prior to calculations. PCA has previously been used to determine flow and relationship between different waters from mining sites (Bäckström and Sartz 2016).

## Results

### Composition of the shale

The mineralogic composition of the shale ash samples A and B and of the fine sample C, representing unprocessed shale, is given in Table 4 (crystalline phases, neglecting the organic fraction). Major mineral components in the fines are the silicates (quartz, feldspars and clay minerals), totally some 73% of the inorganic mass. The transformation of iron in the pyrite and marcasite in the fines into goethite, hematite and jarosite in the shale ash as a result of the pyrolysis is evident.

**Table 4:** Mineral composition of shale ash and fines representing the relative concentrations of crystallized phases normalized to 100% (including data from Åhlgren et al. 2018)

Mineral	Shale ash A	Shale ash B	Fines C
Goethite	5.9	8.9	< 1
Hematite	11.2	17.5	< 1
Jarosite	4.6	3.3	< 1
Gypsum	19.2	1.4	13
K-feldspar	18.2	22.5	15.5
Pyrite	< 1	< 1	12.1
Quartz	34.2	42.8	29.8
Illite/muscovite	6.0	3.6	16.9
Kaolinite	< 1	< 1	6.5
Plagioclase	< 1	< 1	4.4
Marcasite	< 1	< 1	1.8
Alunite	0.7	< 1	< 1

Element concentrations in fines and shale ashes from the deposit and in shale and limestone from the exposed outcrop (N, Fig. 1b) and from the exploration drill core are given in Table 5. The differences between the two shale ashes are significant (S, Fe and Ca among the major components) as well as the differences between the four shale samples (two fines and two alum shales; the same elements). However, concentrations in the shale vary in general between different layers (Andersson et al. 1985). Also, weathering and leaching of shale that is exposed in outcrops would lead to redistribution of elements and changes in composition (Chi Fru et al. 2016). Thus, the observed variations between the shale samples may reflect different locations, as well as differences in weathering state.

**Table 5:** Chemical composition of the solid samples from the waste deposit

Conc. (mg/kg)	Shale ash A	Shale ash B	Fines C	Fines (*n* = 7)	Alum shale (*n* = 5)	Alum shale Drill cores (*n* = 3)	Limestone (*n* = 2)
S	32,300	5400	78,900	42,500	27,500	56,500	13,300
Al	71,500	83,100	61,900	57,200	49,000	56,700	16,900
Mn	232	465	232	155	103	207	736
Fe	83,200	101,000	55,900	35,700	31,000	48,100	15,300
Li					16.4^a^		
Na	816	1410	2670	1780	2570	1100	1300
K	33,600	37,800	25,900	30,900	32,100	29,800	6470
Mg	3260	4040	4460	3900	3800	5200	3920
Ca	29,200	6720	23,500	16,700	4100	9200	323,000
Sr	127	144	85.9	80	84	73.6	184
Cd	0.59	0.70	6.4	1.3	0.36	1.14	0.04
Co	7.30	6.70	26.1	7.5	2.9	23.3	6.35
Ni	51.1	41.6	178	49	26.6	121	8.05
Cu	100	70.2	262	74	40.4	137	11.8
Zn	30.0	33.0	250	78	29.6	63	12
V	661	753	423	499	478	562	20
As	113	155	79.9	54	38.3	65.7	10.6
Mo	213	160	133	136	170	163	1.6
U	200	162	240	84	82	145	0.71

Fines, shale ash (marked C, A and B on the map in Fig. 1d), shale and limestone from the outcrop at the pit lake Norrtorpssjön (location marked N on the map in Fig. 1b) and alum shale exploration drill cores from Norrtorp.

^a^Average of 3, from Allard et al. 2011

It is clear from the total concentrations in drill cores and shale ashes that processing of the shale varied and was incomplete in some cases. Concentrations of sulfur are for instance still quite high in shale ash A compared to shale ash B (Table 5). Sulfur is expected to be lost as SO_2_ during the pyrolysis process. Some trace elements could also be expected to be lost during the process, for instance, zinc that has a low boiling point (907 °C) and therefore could be volatilized during pyrolysis (Saqib and Bäckström 2014). Concentrations of zinc are lower in the ashes compared both to fines and drill core shale but on the same level as in the weathered shale from the pit site (Table 5).

### Groundwater chemistry

Shale residues have been deposited on the same site since the early 1940s (see Fig. 1c). Surface waters, as well as local shallow groundwaters, must have been contaminated by shale leachates already since that time. There was, however, no regular monitoring of surface water or groundwater quality in the area until 1993, when monitoring started in the small stream north of the deposit, and not until 2003–2004 in groundwaters in the vicinity of the deposit (from wells G1–G10).

#### Major components

**Fig. 2:**
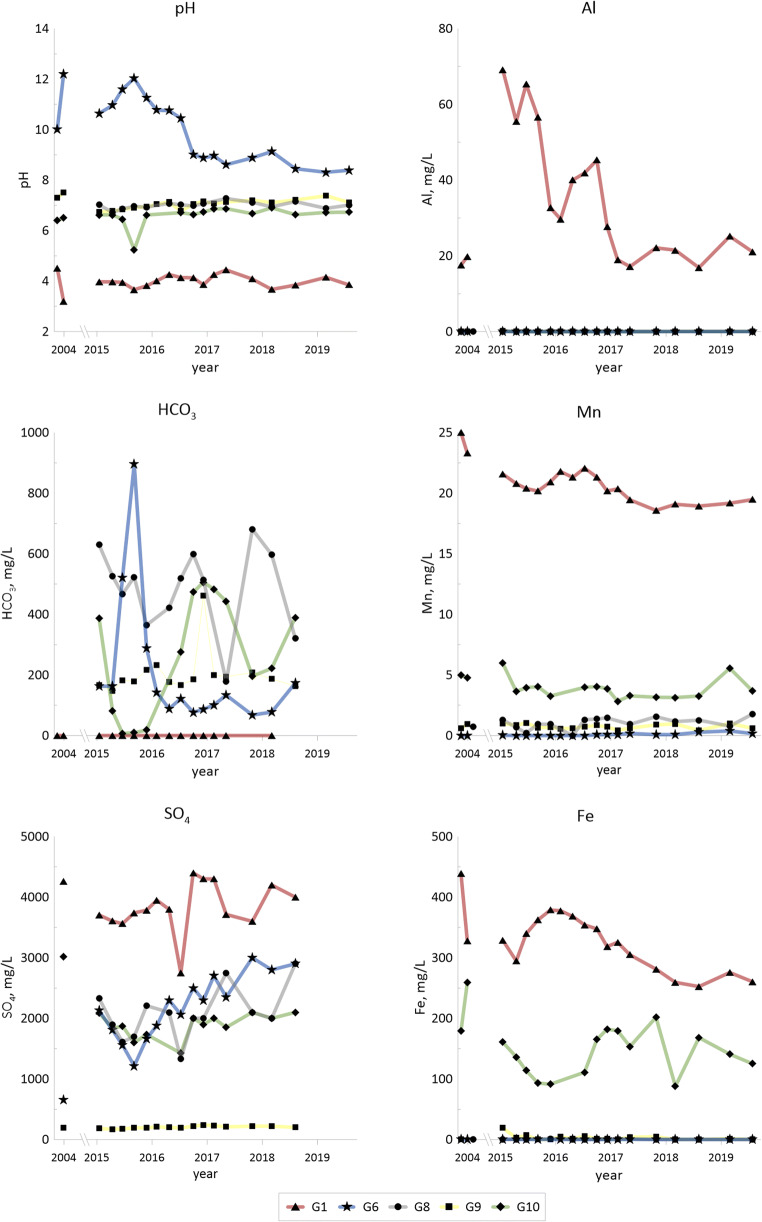

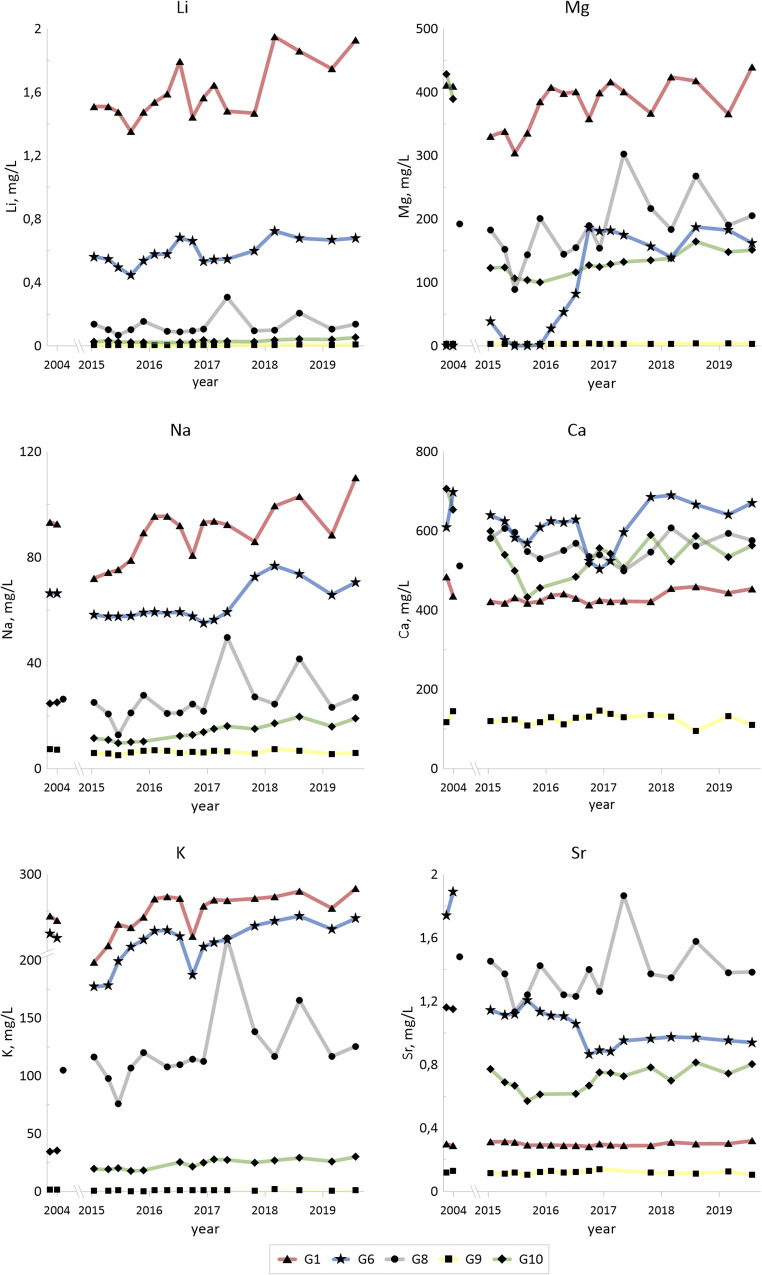
*Temporal changes for pH, concentrations of hydrolysable elements (Al, Mn* and *Fe) and major anions* (*HCO*_*3*_ and *SO*_*4*_*) in selected groundwater samples for 2004 (according to* Holm et al. 2005*) and for 2015*–*2019.* Alkali metals (Li, Na and K) and alkaline earth metals (Mg, Ca and Sr)

Concentrations of major components (cations: Na, K, Mg, Ca, Al, Mn and Fe; also Li and Sr; anions: HCO_3_^−^ and SO_4_^2−^; pH) from 2004 (Holm et al. 2005) and from 2015–2019, are given for selected groundwaters of different types (G1, G6, G8, G9 and G 10) in Fig. 2, which illustrates the variation of concentrations with time during the period 2004–2019.

All measurements during the period for all 11 wells G1–G11 are summarized in Table 6: median and min–max values for the sampling period 2015–2019, as well as median values from 2004 (Holm et al. 2005).

**Table 6:** Concentrations of major components (mg/L) and pH in G1–G11 water: for each element, the first row represents median value 2015–2019, second row represents min and max values 2015–2019 and third row represents median values 2004

	G1 (*n* = 17) (2015–2019)	G2 (*n* = 17)	G3 (*n* = 17)	G4 (*n* = 11)	G5 (*n* = 16)	G6 (*n* = 17)	G7 (*n* = 17)	G8 (*n* = 15)	G9 (*n* = 17)	G10 (*n* = 15)	G11 (*n* = 17)
Na	91.9	131	110	71.2	71.9	59.0	11.4	24.5	6.19	13.9	13.2
	72.1; 110	116; 211	86.9; 133	60.6; 80.3	65.9; 77.8	55.2; 76.8	10.7; 18.0	12.7; 49.7	4.98; 7.43	9.64; 19.8	11.4; 20.4
	92.9	133	32.9	32.1	45.3	66.4	17.7	26.4	7.32	24.8	–
K	265	7.08	340	172	118	211	3.65	116	1.07	25.0	10.3
	198; 281	5.05; 7.88	252; 420	136; 198	99; 129	177; 243	2.27; 5.01	75.7; 213	0.09; 2.02	17.7; 30.0	8.49; 12.9
	240	10.7	62.3	45.9	72.4	216	8.87	105	1.61	35.0	–
Mg	398	0.28	456	260	96.2	139	0.14	183	3.13	127	12.7
	305; 440	0.20; 6.9	337; 506	222; 280	83.7;107	0.11; 187	0.095; 0.25	88.6; 303	2.50; 3.57	100; 164	10.1; 18.2
	410	0.09	61.4	48.3	33.2	0.09	0.52	192	3.02	409	–
Ca	425	64.1	465	414	416	624	31.5	561	128	534	321
	414; 459	55.7; 161	447; 484	376; 425	373; 456	503; 690	22.2; 38.7	499; 607	94.4; 146	433; 599	239; 406
	460	88.9	537	262	287	654	39.6	511	131	680	–
Al	29.7	0.0083	2.28	0.0112	0.0129	0.0027	0.00198	0.00412	0.0017	0.0018	0.008
	16.9; 69.1	0.0025;0.071	0.50; 5.45	0.00386; 3.096	0.00698; 0.0256	0.00113; 0.039	0.0013; 0.0585	0.00145; 0.134	0.0012; 0.181	0.001; 0.0410	0.00185; 0.203
	18.7	0.17	1.87	0.017	0.00268	0.0058	0.138	0.0138	0.002	0.0142	–
Mn	20.4	0.0067	13.9	9.3	4.2	0.064	0.198	1.21	0.709	3.67	1.91
	18.6; 22.1	0.0003; 0.45	10.3; 16.1	7.28; 9.93	3.32; 4.49	0.00017;0.38	0.13; 0.365	0.199; 1.78	0.43; 1.04	2.83; 5.96	0.984; 3.77
	24.2	0.0004	5.18	3.75	1.87	0.0028	0.0012	0.71	0.765	4.87	–
Fe	326	0.0267	107	95	13.3	0.047	1.36	0.239	1.275	141	3.31
	252; 379	0.0024; 3.3	63; 167	19.8; 157	2.5; 17.3	0.0021; 0.468	0.0026; 3.49	0.018; 1.37	0.0074; 19.1	87.9; 202	0.192; 13.7
	384	0.0272	13.7	0.58	0.11	0.04	0.049	0.0066	0.118	219	–
Cl^−^	38	42.6	54.0	42	12	19	12.9	11	6.4	7.65	22
	29; 93	32.0; 49.1	24.6; 90.2	39; 60	11; 29	16; 63	9.50; 15.0	10; 60	5.8; 10.6	7.5; 9.0	17; 26
	–	–	–	–	–	–	–	–	–	–	–
SO_4_^2−^	3785	382	3822	2280	1500	2300	68.0	2000	200	1900	518
	2746; 4400	287; 800 308	3400; 4300	1829; 2700	1300; 2577	1215; 3000 656	35.7; 110	1336; 2900	171; 240	1427; 2100	358; 950
	4260		1720	869	476		23.0	–	196	3020	–
HCO_3_^−^	0	31	127	180	472	133	31.3	518	185	275	394
	0	13.2; 78.1	0; 195	108; 318	416; 600	67.9; 897	9.5; 45.7	178; 681	147; 462	7.32; 506	221; 548
	0	170	0	74	695	1400	210	–	225	465	–
pH	3.97	8.77	6.1	6.63	6.06	9.13	6.81	7.01	7.07	6.67	6.65
	3.66; 4.44	6.89; 10.0	5.82; 6.42	6.49; 6.81	5.90; 6.26	8.31; 12.0	6.47; 7.88	6.72;7.27	6.74; 7.38	5.24; 6.90	6.21; 6.88
	3.48	10.70	3.82	6.55	6.50	10.3	10.40	–	7.39	6.45	–

Variations in water chemistry between the groundwater wells are substantial, e.g., with pH ranging from 3.7 to 12 (from 3.2 to 12.2 in 2004), total carbonate from 0 to 900 mg/L and sulfate from 36 to 4400 mg/L during the sampling period. G1 has the lowest pH and is evidently highly affected by shale leachates with high concentrations of sulfate and iron, as well as trace elements (see below) throughout the entire time period.

Redox conditions for the groundwaters indicate two groups: G2, G6 and G8 (average ferrous iron concentration 0.2 mg/L and average redox potential 320 mV) and G1, G3, G4, G5, G7, G9, G10 and G11 (average ferrous iron concentration 8.6 mg/L and average redox potential 190 mV). The larger group is somewhat more reducing as indicated by both the redox potential and the dissolved ferrous iron concentrations.

From Table 6, and considering the depth and positions of the wells and the various layers around and below the wells (Table 1), it can be assumed that G2 and G7 may represent deep groundwater from the sandstone layer while G9 and G10 may represent shallow groundwater from the till surrounding the deposit. No alum shale waste was deposited nearby the G9 site, and the ground does not seem to be reworked since it is covered by undisturbed quaternary deposits (till). It is therefore assumed that G9 may represent local background conditions. Water from G10, however, also from the till horizon, appears to be affected by shale leachates as indicated by higher levels of major components (sulfate, potassium and magnesium in particular) and trace metals (Sr, Li, Co and Ni) and with slightly lower pH.

#### Trace elements

**Fig. 3:**
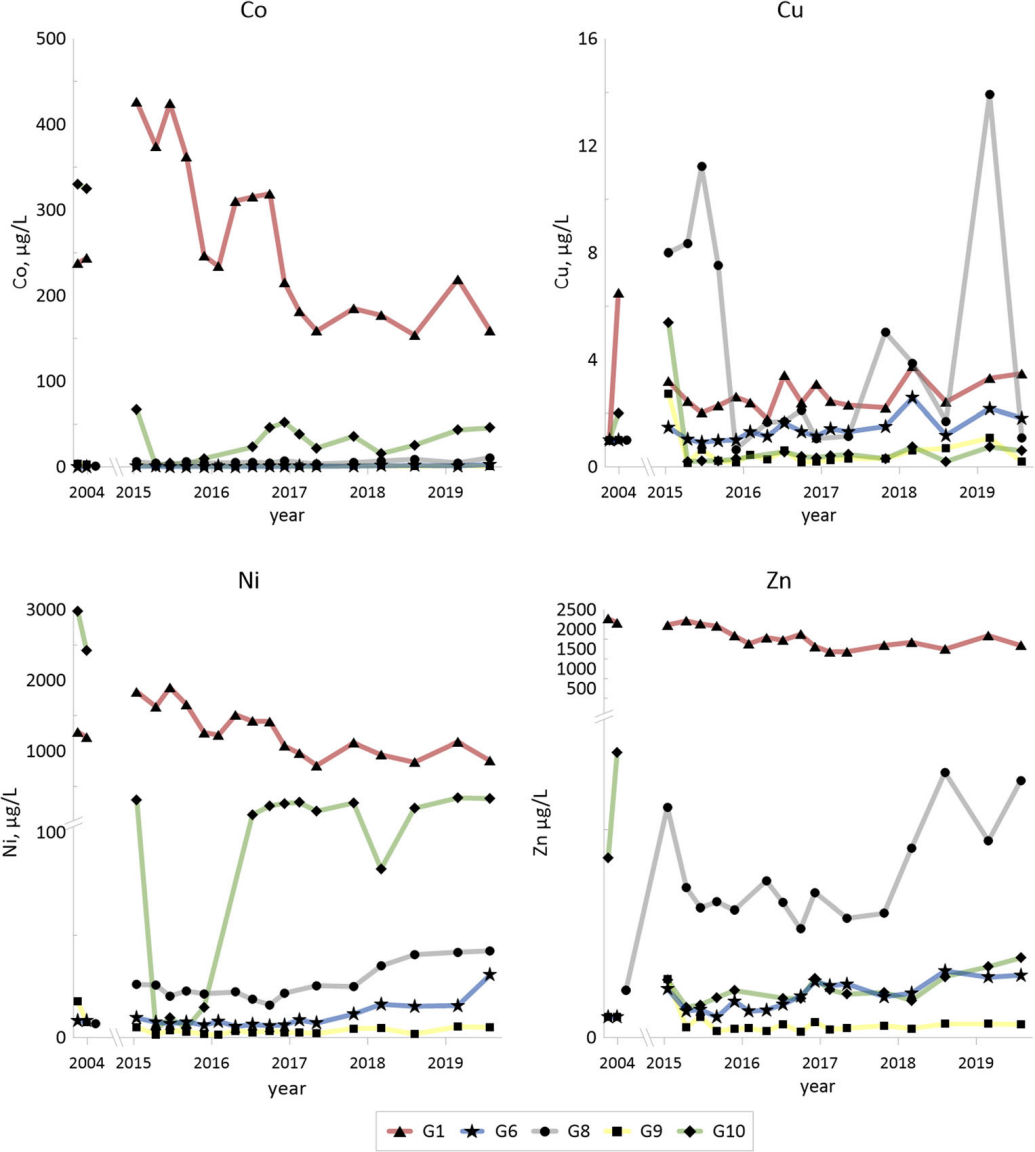

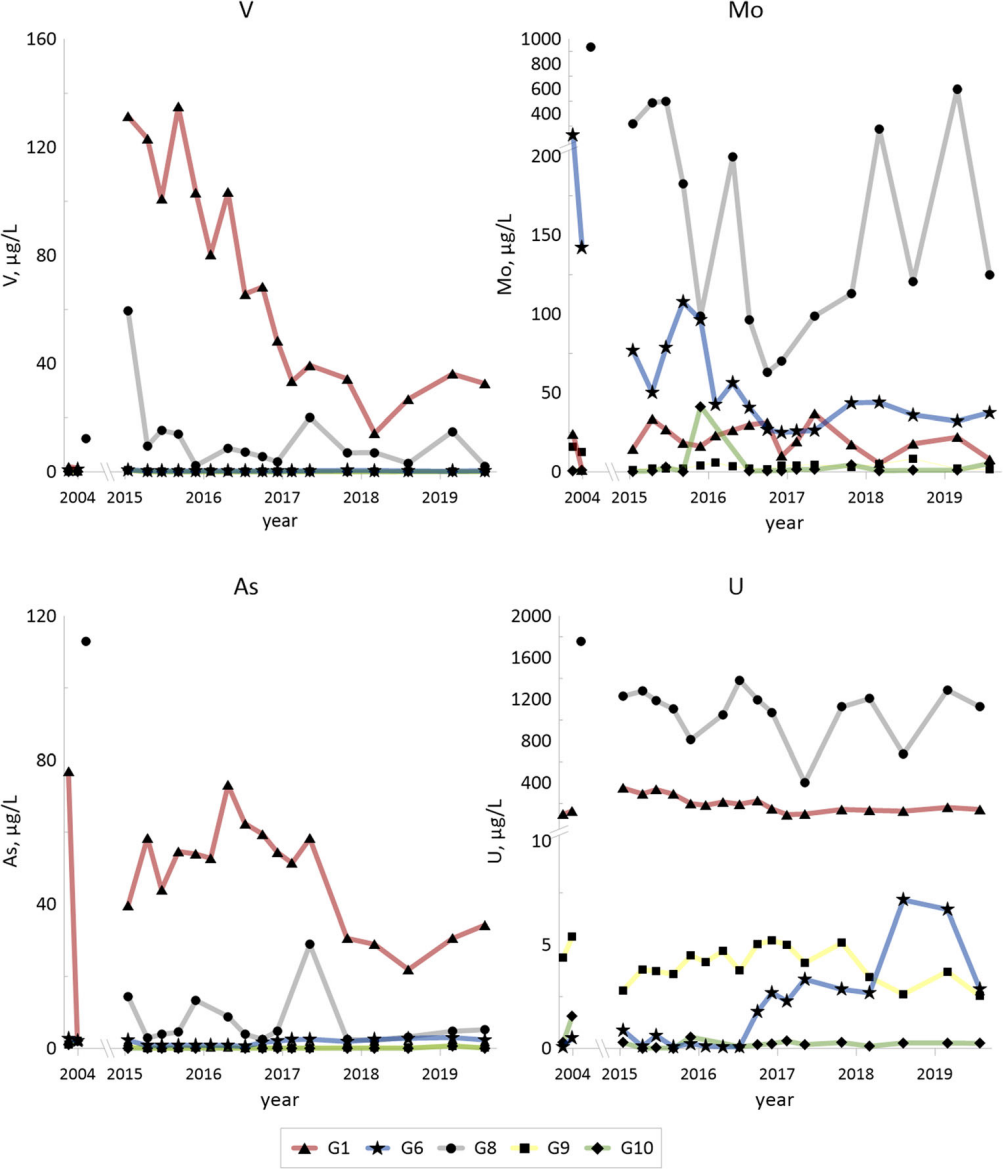
Temporal changes for hydrolysable trace metals (Co, Cu, Ni and Zn) in selected groundwater samples for 2004 (according to Holm et al. 2005) and for 2015–2019. Redox sensitive trace elements (V, As, Mo and U)

Concentrations of some of the trace elements (Co, Ni, Cu, Zn, V, As, Mo and U; also Li and Sr in Fig. 2) from 2004 and from 2015–2019 are given for the selected groundwaters (G1, G6, G8, G9 and G10) in Fig. 3.

Concentrations of the trace elements are summarized in Table 7 (as in Table 6): median and min–max values for the sampling period 2015–2019, as well as median values from 2004.

**Table 7:** Concentrations of trace elements (μg/L) in G1–G11 water: for each element, the first row represents median value 2015–2019, second row represents min and max values 2015–2019 and third row represents median values 2004

	G1 (*n* = 17)	G2 (*n* = 17)	G3 (*n* = 17)	G4 (*n* = 11)	G5 (*n* = 16)	G6 (*n* = 17)	G7 (*n* = 17)	G8 (*n* = 15)	G9 (*n* = 17)	G10 (*n* = 15)	G11 (*n* = 17)
Cd	6.06	0.005	0.85	0.03	0.03	0.043	0.004	0.76	0.01	0.02	0.08
	4; 15	0.002; 0.1	0.27; 4.2	0.025; 1.45	0.02; 0.12	0.02; 0.15	0.001; 0.1	0.097; 1.49	0.002; 0.1	0.002; 0.05	0.003; 0.65
	6	0.05	1.35	0.08	–	0.30	0.05	0.31	0.05	0.08	–
Co	234	0.1	35.9	0.53	0.65	0.66	0.09	5.12	1.2	25.3	0.87
	154; 427	0.04; 0.49	12.7; 87.1	0.32; 9.3	0.47; 1.65	0.46; 2.73	0.02; 0.16	1.7; 10.9	0.56; 2.0	1.1; 66.7	0.30; 6.94
	241	0.05	43.7	0.77	0.25	0.09	0.05	0.78	2.90	328	–
Ni	1224	0.93	253	20.3	4.8	7.6	0.57	24.9	2.92	191	6.69
	798; 1897	0.48; 5.10	107; 541	16.6; 54.7	3.15; 15.8	5.5; 30.6	0.097; 1.83	16.0; 42.3	1.30; 5.3	3.85; 339	2.3; 51.2
	1230	0.50	227	47.6	1.53	8.18	0.50	6.95	12.6	2700	–
Cu	2.44	2.82	5.47	1.80	1.59	1.32	0.34	2.12	0.31	0.39	0.41
	1.79; 3.75	1.35; 6.09	2.2; 20.4	1.09; 60.9	0.82; 3.89	0.98; 2.58	0.21; 1.04	0.65; 13.9	0.14; 2.73	0.19; 5.4	0.27: 5.31
	3.75	1.0	10.4	1.33	1.00	1.0	1.22	1.0	1.0	1.5	–
Zn	1727	2.82	675	3.92	7.28	1.98	0.51	6.97	0.50	2.18	2.55
	1430; 2211	0.18; 12 1.0	247; 4740	2.13; 110	5.30; 11.6	0.98; 3.20	0.16; 1.67	5.23; 12.8	0.28; 2.80	1.46; 3.83	0.58; 26.2
	2200		282	4.71	5.43	1.0	1.0	2.26	1.0	11.2	–
Sr	293	1670	364	382	1241	975	287	1374	119	728	384
	284; 320	1532; 2788	352; 401	330; 408	1096; 1381	865; 1205	240; 366	1135; 1867	104; 141	572; 815	242; 464
	294	2250	221	247	682	1815	1070	1480	125	1155	–
Li	1536	58.8	2136	1052	609	578	12.3	102	5.2	26	14.1
	1352; 1949	41.0; 161	1871; 2559	807; 1181	506; 697	445; 724	9.3; 19.6	69; 306	4.2; 7.8	21; 52	10.4; 27.6
	–	–	–	–	–	–	–	–	–	–	–
V	65.8	0.01	0.45	8.98	0.66	0.34	0.006	7.34	0.01	0.04	0.62
	14.3; 135	0.004; 0.16	0.15; 16.6	2.31; 105.8	0.35; 1.83	0.24; 0.70	0.003; 0.36	2.13; 59.4	0.003; 0.6	0.01; 0.64	0.21; 2.56
	1.67	1.8	0.97	8.53	0.17	0.70	2.01	12.3	0.05	0.08	–
As	52.8	0.52	86.8	1.49	0.91	1.91	0.06	4.5	0.13	0.05	4.95
	21.9; 73.1	0.18; 2.86	37.0;172	0.16; 65.9	0.52; 1.78	0.55; 3.02	0.02; 1.04	2.20; 28.9	0.08; 0.7	0.03; 0.7	1.14; 28.1
	39.5	4.0	13.2	4.50	1.07	2.39	4.0	113	1.5	1.5	–
Mo	19.3	1.90	405	46.9	37.3	42.3	1.41	125	2.94	0.93	32.1
	5.21; 36.7	0.74; 3.01	172; 633	24.9; 413.8	30.5; 59.7	24.9; 108	0.50; 4.58	63; 595	1.15; 8.22	0.19; 41.3	2.5; 78.4
	12.6	7.50	4.33	30.7	50.7	186	2.04	935	14.1	0.75	–
U	183	0.06	40.0	89.5	2.48	1.77	0.025	1126	3.82	0.23	26.4
	93.8; 349	0.008; 1.20	9.0; 72.2	41.9; 154	1.89; 10.2	0.07; 7.16	0.001; 0.21	400; 1380	2.52; 5.22	0.013; 0.55	3.1; 65.0
	113	0.01	16.3	8.47	16	0.30	0.01	1760	4.90	0.93	–

**Fig. 4:**
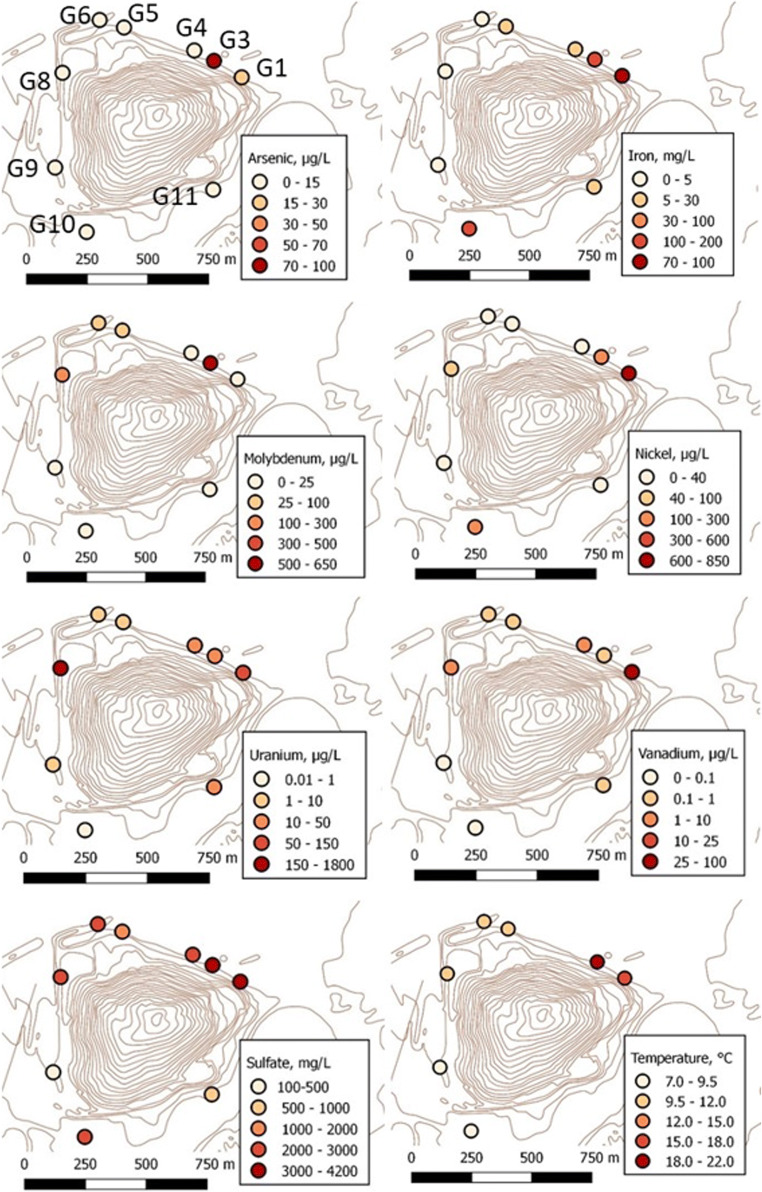
Spatial distribution of concentrations for As, Fe, Mo, Ni, U, V and SO_4_^2−^ in groundwaters (except deep groundwater) around the waste deposit (cf. Fig. 1d). Concentration levels from October 2018 and water temperatures at 1-m depth from November 2017 (no temperature data available for G4 and G11)

The large variation of concentrations between wells G1 and G11 for some of the trace elements and sulfate is further illustrated in Fig. 4, with concentration levels from October 2018. Highest levels (median values, 2015–2019) in groundwaters G1–G11 are obtained in G1 (with the lowest pH in the series) for Cd, Co, Ni, Zn and V, and the highest levels for Cu, Li, As and Mo are obtained in G3. Levels from the measurements in 2018 largely confirm this general trend (Fig. 3). The highest uranium levels are obtained in G8, in 2004, 2015–2019 and 2018. The differences in temperatures indicate that the deposit is hot, primarily in the north-eastern part (G1 and G3) and to some extent also in the north-western part (G5, G6 and G8).

### Groundwater classification

#### Chemistry profiles

Analysis of the chemistry of groundwaters G1–G11 (pH, major components and trace elements, Figs. 2 and 3 and Tables 6 and 7) and combining with data on the location and positions of the wells (Fig. 1d and Table 1), as well as data on the composition of the shale waste categories (Table 5) may be the basis for classification of the groundwaters (cf. discussion under Results).

*Groundwaters G2 and G7* come from the lower aquifer, in the sandstone layer. G7 seems to be unaffected by shale leachates from the deposit. G2 has higher levels of sulfate, lithium and strontium as well as higher pH, up to 10 or above in 2018 and in 2004, giving lower levels of manganese and iron. Alkaline pH may, however, indicate contact with cement used to seal the contact between the two aquifers, not observed in G7.

*Groundwaters G6, G9 and G10* come from the upper aquifer, in the till layer. G9 seems to be largely unaffected by leachates from the deposit. The concentrations show a good correspondence with national values for unaffected groundwater where the span for the 5th to 95th percentile for, e.g., arsenic is 0.03–0.92, cobalt 0.01–1.2 and nickel 0.07–4.0 μg/L. Sulfate (median 200 mg/L in G9 compared to 45 mg/L) and iron (median 1.28 mg/L in G9 compared to 1.1 mg/L) surpass the 95th percentile for unaffected groundwater (SGU 2013). Median pH for G9 is 7.09. There is a possibility of natural presence of shale and pyrite in the till, which could explain the elevated sulfate concentrations that reflect the general background conditions at the Kvarntorp site.

*Groundwaters G1 and G3* from the upper aquifer are clearly distinguished from the other groundwaters. Both have high sulfate, nickel and lithium concentrations and low pH indicating a progressing weathering of the shale waste. G1 has the water with the highest concentrations of iron (median 326 mg/L) and sulfate (median 3785 mg/L) and with the lowest pH during 2015–2019 (below 4). In 2004, also G3 was acidic (pH 3.6–4.3), but during 2015–2019, pH was higher, in the range 5.82 to 6.42. Pyrite containing shale and fines are expected to generate acidic leachates and subsequent metal releases, as documented in numerous laboratory tests and measurements in the field at shale mining sites (e.g., Bäckström 2010; Allard et al. 2011; Karlsson et al. 2012; Wilke et al. 2015; Chi Fru et al. 2016; Åhlgren et al. 2020; Sartz et al. in preparation). The data from G3 might indicate that the pyrite weathering has decreased, but increased electrical conductivity and higher sulfate and iron concentrations contradict this. Also, potassium and magnesium concentrations are higher in 2015–2019 compared to 2004. The pH increase may possibly be due to illite buffering, as has been reported elsewhere (Puura 1998) or carbonate buffering.

*All other groundwaters, G4, G5, G8 and G11*, can be considered till water (G9) from the upper aquifer contaminated with leachates from the shale wastes as indicated by the levels of trace elements from the shale. However, pH is generally above 6.5. This indicates and confirms that weathering of shale ashes, and possibly semi-coke, generates leachates that are not acidic. Weathering is progressing even in the absence of acid generating sulfides, in agreement with observations in laboratory leaching tests in progress (Sartz et al. in preparation). Progressing shale weathering has previously been observed in buffered aquatic systems in the field at the Kvarntorp site, with pH above 7–8 (Allard et al. 2011; Karlsson et al. 2013; Åhlgren et al. 2020).

*Groundwater G6* belongs to the sequence G4, G5 and G8 but with the difference that median pH is 9.13 during 2015–2019 (maximum 12.0) and maximum 12.2 in 2004. There is no cement seal around the well that could be the source of alkalinity. Estonian oil shale ash is rich in free lime and anhydrite and produces alkaline leachates with pH 12–13 (Velts et al. 2011) which is generally not the case for the Kvarntorp shale ash which generally produces a circumneutral or slightly acidic leachate (Karlsson et al. 2012), and G6 is the only shallow groundwater well in Kvarntorp where alkaline conditions have been found. For the Estonian oil shale, it is the high content of limestone in the shale (calcite 44%) which when heated (800 or 1400 °C depending on method) generates calcium oxide and pH above 12 in contact with water (Pihu et al. 2019). The shale used for oil production in Kvarntorp is not as rich in carbonates as the Estonian oil shale. Calcite is not a major mineral component in the shale, but limestone waste was disposed at the Kvarntorp deposit together with the shale residues. The high pH found in G6 may reflect the presence of calcium hydroxide from the decomposition of calcium oxide originating from the degradation of calcium carbonate in the deposit, still burning with temperatures exceeding 500 °C. The pH variation in G2 from the lower aquifer (sandstone; between 6.89 and 10.0), however, could be caused by cement leaching from the sealing material (Table 1). Also G7 might have been influenced by cement resulting in much higher alkalinity and pH in 2004 compared to the period 2015–2019.

*Groundwater G8* has the highest uranium levels, median values above 1 mg/L during 2015–2019. The uranium comes from sludges of possibly processed shale residues that are deposited close to the filter section of the pipe in the well.

#### PCA

**Fig. 5:**
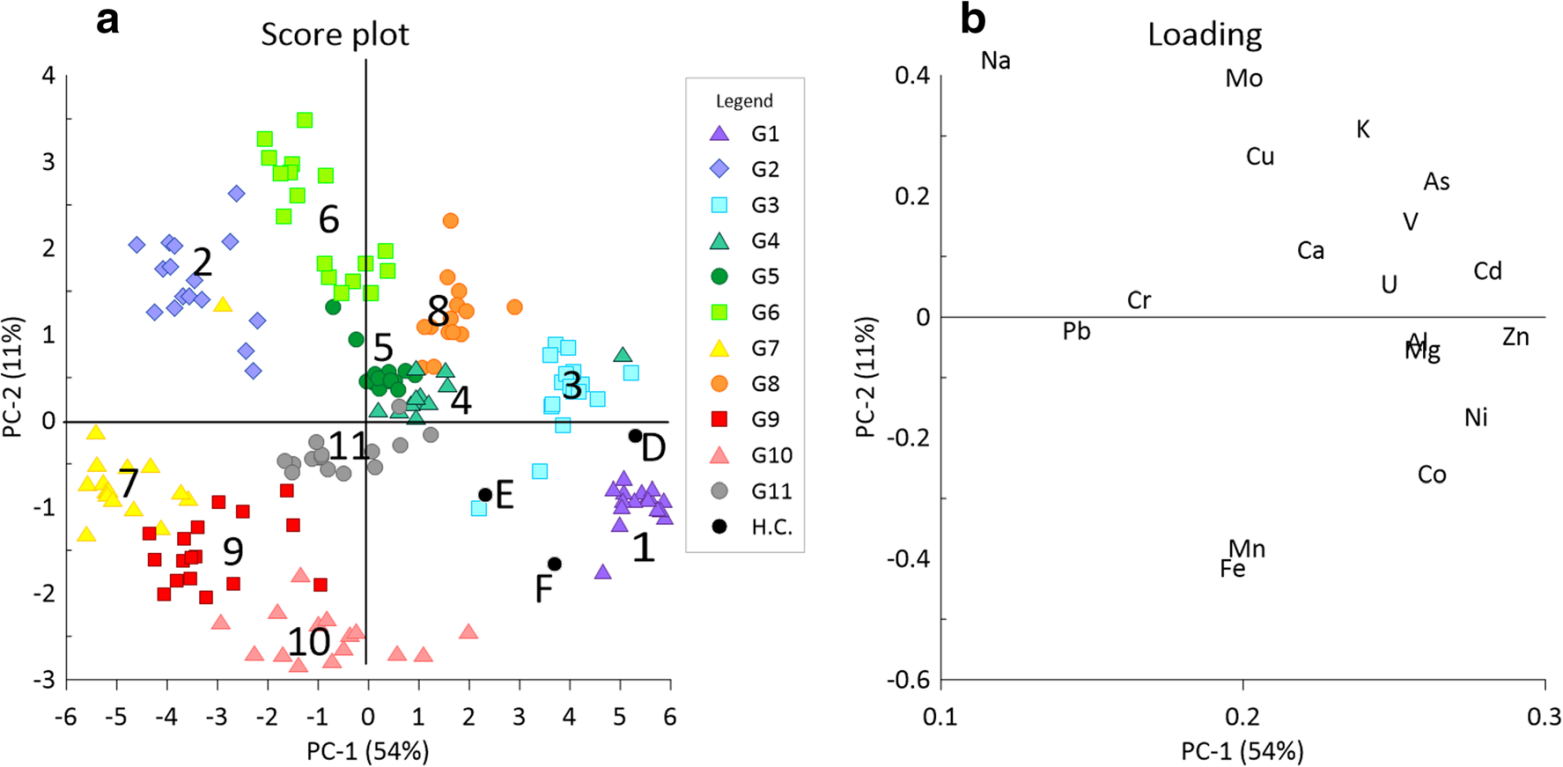
**a** Principal component analysis (score plot) showing all groundwater samples and data from day 7 from the humidity cells (Sartz et al. in prep), where D = fines, E = red shale ash and F = black shale ash. The first two principal components explain 65% of all the variation in the data set. **b** Loading plot showing the relationship between the different elements

A PCA based on data for all groundwater samples, as well as data from humidity cell leaching tests (Sartz et al., work in progress) is given in Fig. 5 (score plot) together with the loading plot showing element relationships.

The PCA score plot confirms the assessments based on the measured concentrations in the groundwaters combined with information on the location and depths of the wells (Fig. 1d and Table 1). Almost all the groundwaters are unique, as illustrated by the score plot, although the G4, G5 and G8 groundwaters are fairly similar in composition. Principal component 1 (PC1, 54%) explains significantly more of the data variation compared to the second principal component (11%). Groundwaters G2 (average PC1 score − 3.43) and G7 (average PC1 score − 4.75) are located in the sandstone aquifer and G9 (average PC1 score − 3.12) is located in the till upstream the waste deposit. These unaffected groundwaters (compared to the other groundwaters in the area) have highly negative PC1 score values. Groundwaters G6 (average PC1 score − 0.93) and G11 (average PC1 score − 0.55) are slightly affected by contaminants and found at some distance from the deposit. Groundwater G10 (average PC1 score − 0.76) is affected by surface run-off from parts of the deposit with fines. Groundwaters G4 (average PC1 score 1.32), G5 (average PC1 score 0.27) and G8 (average PC1 score 1.65) are more closely located towards the deposit and are very tightly grouped together in the PCA. Groundwaters G1 (average PC1 score 5.35) and G3 (average PC1 score 3.89) are located towards the right (high PC1 scores) in the PCA plot and close to fines and black ash from the humidity cell experiments (D and F). This indicates a significant impact from the waste materials on these groundwaters. Groundwater G1 is closest to fines while G3 is found closer to shale ashes (black and red) in the deposit. Groundwater G1 is thus the most affected groundwater positioned straight north of the fines located along the eastern side of the waste deposit (Fig. 1d). This confirms the fact that G1 is affected mainly by leachates from fines, even if it is not possible, solely from these data, to determine the exact proportion between fines leachates and black/red shale ash leachates. Score values from PC1 are thus tentatively interpreted as describing the input of contaminated leachates from the shale waste in the deposit. This is confirmed by the loading plot (Fig. 5b) having high values for nickel and uranium in PC1. PC2 is probably governed by redox reactions as dissolved iron and manganese are found with strongly negative loading values in PC2. This is also strengthened by the fact that groundwaters G2, G6 and G8 with more oxidizing conditions have positive scores in PC2 (Fig. 5a). There is a clear relationship between the average score values for PC1 and logarithmic nickel concentrations (*r*^2^ 0.77) and logarithmic uranium concentrations (*r*^2^ 0.64) indicating that PC1 is a good proxy for the level of contamination in the groundwaters. This clearly shows that the combination of PCA and humidity cell experiments can provide information about what materials affect the different groundwaters. PCA has been used with success in other groundwater systems to identify contamination sources (Sappa et al. 2014; Rao 2014; Bäckström and Sartz 2016). In total, the PCA confirms the groundwater classifications assessed solely from the monitoring data (water chemistry) combined with data on the wells (location, depths etc.).

## Discussion

### Element distribution and speciation

Figs. 2 and 3 (concentrations of major elements and trace elements in selected groundwaters, 2015–2019), Tables 6 and 7 (concentrations of all of the elements in all of G1–G11, median and max–min values, 2015–2019) and Table 5 (composition of the solid materials, shale ash and fines from the deposit, shale from the pit lake area) illustrate and define the overall distribution and exchange of elements between solid materials and groundwater in the shale waste/groundwater system that constitutes the Kvarntorp deposit. Most important chemical parameter in this system that largely governs the leaching/weathering of the solid materials and concentrations and element speciation in the groundwater is pH (Table 6 and Figs. 2 and 3).

The groundwater G9 from the upper aquifer in the till is dominated by Ca–HCO_3_–SO_4_ with pH around 7.07 and total concentrations of dissolved solids around 530 mg/L (median values, Table 6). The groundwater G7 from the lower aquifer in the sandstone is also dominated by Ca–HCO_3_–SO_4_ with pH around 6.81 and concentrations of dissolved solids around 160 mg/L (median values, Table 7). The location of the G9 well is upstream the deposit. There is no significant change in chemistry in the water during the period 2004–2019 and no evident impact of contamination from shale leachates. Thus, G9 may serve as a background and reference for the assessment of the impact of leachates on the other groundwaters except G7 and G2. There is no apparent contamination of elements from the shale in the G7 water. This indicates that the green shale layer between the deposit and the sandstone horizon 31 m below the surface prevents exchange of water between the upper aquifer in the till/shale waste and the lower aquifer in the sandstone.

#### Major components—Na, K, Mg, Ca, HCO_3_^−^, SO_4_^2−^, (Cl) and pH

All shallow groundwaters, with the exception of G9, exhibit significant contributions of shale leachates, affecting to various extent all of the measured parameters. The highest contributions of leachates and largest effects on the concentrations of major elements and sulfate, as well as on pH, are observed in G1 (lowest pH), followed by G3 (Fig. 2 and Table 6).

The high pH in G6, most likely from calcium hydroxide originating from degraded calcium carbonate at high temperature (see discussion under Chemistry profiles), is decreasing with time from 12 in 2015 to 8 in 2019, indicating a depletion of calcium hydroxide but possibly equilibrium with calcium carbonate.

**Fig. 6:**
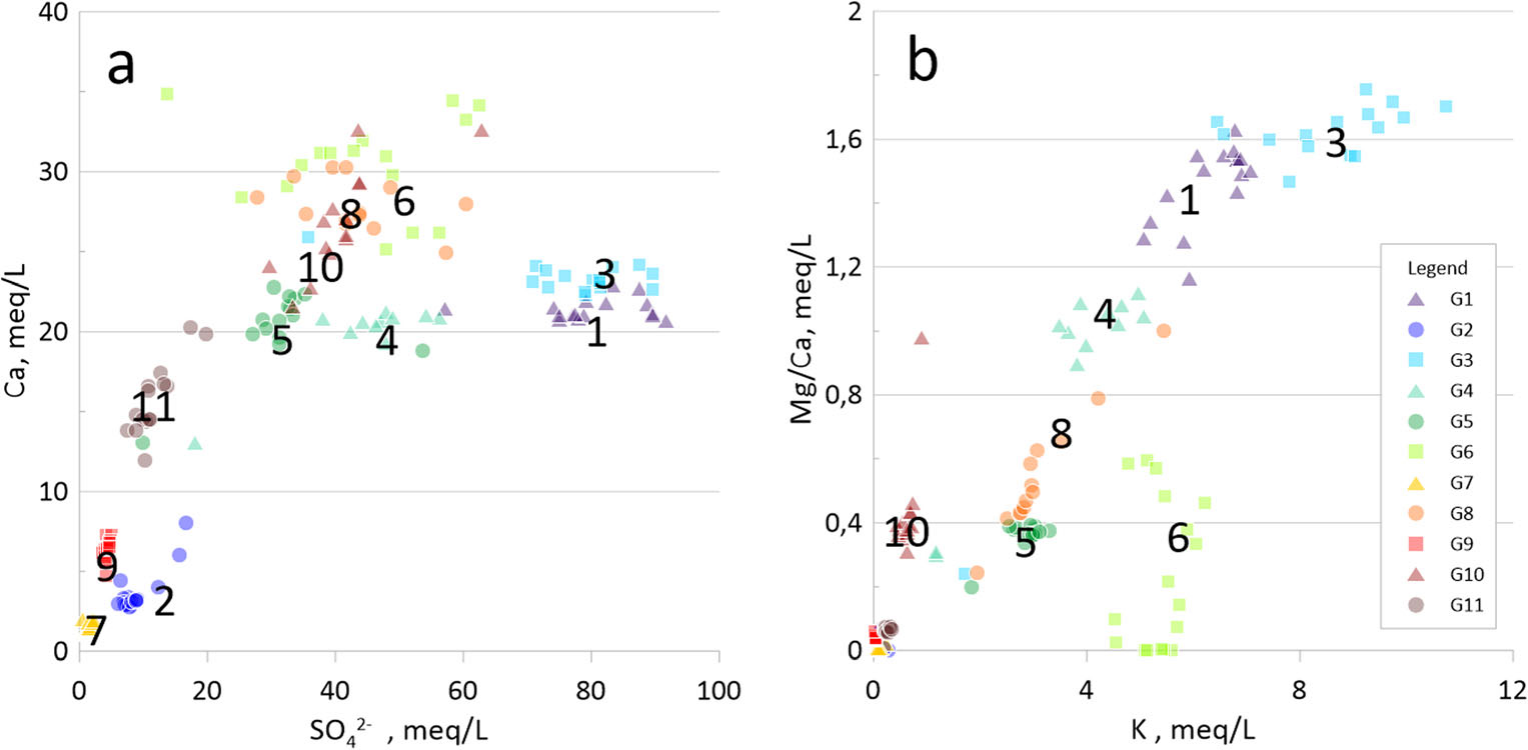
**a** Calcium concentrations (meq/L) versus sulfate concentrations (meq/L) in groundwaters G1–G11. **b** Magnesium/calcium ratios versus potassium concentrations (meq/L) in groundwaters G1–G11

G1 and G3 differ from the other groundwaters when calcium concentrations are plotted against sulfate (Fig. 6a). Calcium concentrations are roughly only half of what would be expected compared to the trend for the other groundwaters. This pattern might indicate that there are no carbonates enough in the vicinity of G1 and G3 to buffer the acid being generated, which is consistent with leaching tests from Degerhamn where calcium was less present in acidic samples than in non-acidic lime-burned shale ash with dissolution of gypsum (Lavergren 2008). Calcium removal from the samples is likely due to precipitation of gypsum (saturation index − 0.14 and − 0.04 for G1 and G3, respectively) at high sulfate concentrations.

Mg/Ca ratios plotted against potassium concentrations (Fig. 6b) provide information about ongoing redox activities in the deposit. A low Mg/Ca ratio suggests (1) that no magnesium bearing lime reacts, (2) that magnesium has been washed out or (3) that the calcium concentrations are limited by gypsum equilibrium. There are two possible reasons for lime not to react. Either there is no formation of sulfuric acid or all lime is consumed (Puura 1998). G1, G3 and G4 (located immediately downstream the waste deposit) display Mg/Ca ratios close to 1 or greater which is an indication of redox activities. These groundwaters are slightly reducing, though, with ferrous iron concentrations between 6 and 10 mg/L indicating that oxidizing reactions take place in the deposit upstream the groundwater wells. More reducing conditions close to the wells will, however, reduce the possibility for secondary immobilization when iron oxyhydroxides are being dissolved. Weathering of illite generates potassium in a slow process. It has been suggested that monitoring of potassium concentrations in combination with magnesium and calcium could provide information about the development in the deposit (Holm et al. 2005). A decreasing Mg/Ca ratio combined with an increasing potassium concentration would indicate a decrease in lime buffering and ongoing sulfuric acid production being buffered by illite instead. No such trends can be distinguished in the time span 2004–2019. There is a correlation (*r*^2^ 0.72) between the Mg/Ca ratio and the potassium concentration when all 11 locations are included and an even stronger correlation (*r*^2^ 0.94) when G6 and G10 are excluded. For G6, the Mg/Ca ratio is lower than for other wells with similar potassium concentrations. For G6, this could be due to equilibrium with brucite (saturation index − 0.02) since G6 is the only groundwater in equilibrium with brucite (see below). G10 displays the opposite relationship between Mg/Ca and potassium compared to G6, but since 2004, G10 has approached the linearity between Mg/Ca and potassium shown by the other groundwaters. This is an indication of decreased weathering at this site upstream the deposit, since also the electrical conductivity has decreased. For G3 and G4, both the Mg/Ca ratio and the potassium concentrations have increased since 2004 suggesting that weathering have increased in these waters. This is further strengthened by an increase in electrical conductivity in 2015–2019 compared to 2004. From Mg/Ca ratios, no general conclusion can be drawn for the entire area about changing weathering. Instead, a suggestion is that weathering rates are decreasing at some parts and increasing at other.

#### Solubility control

SI from the geochemical calculations for the groundwaters are given in Table 8. Positive SI (> 0.5) indicates oversaturation, negative SI (< 0.5) indicates undersaturation and SI in the range − 0.5 to 0.5 is considered to be in equilibrium with the considered solid phase. Solid phases likely found at equilibrium with the groundwaters are calcite, celestite, dolomite, gibbsite, gypsum, jarosite and siderite. The presence of jarosite and gypsum has been confirmed to exist in the different waste materials within the waste deposit (Table 4).

**Table 8:** Selected saturation indices (SI) for possible solid phases (hydroxides, sulfates and carbonates) in groundwaters from G1–G11

		G1	G2	G3	G4	G5	G6	G7	G8	G9	G10	G11
AlOHSO_4_		0.61	− 5.5	***0.37***	− 3.1	− 1.8	− 14	− 4.2	− 3.8	− 4.1	− 3.4	− 2.8
Alunite	KAl_3_(SO_4_)_2_(OH)_6_	4.8	− 4.6	11	2.5	2.4	− 19	− 3.8	− 1.0	− 4.0	− 1.3	***0.04***
Anhydrite	CaSO_4_	***− 0.43***	− 1.6	***− 0.30***	***− 0.46***	***− 0.51***	***− 0.26***	− 2.3	***− 0.44***	− 1.4	***− 0.46***	− 1.0
Gypsum	CaSO_4_:2H_2_O	***− 0.14***	− 1.3	***− 0.04***	***− 0.22***	***− 0.19***	***0.07***	− 1.9	***− 0.11***	− 1.1	***− 0.13***	− 0.68
Brucite	Mg(OH)_2_	− 12	− 7.6	− 6.9	− 5.8	− 8.6	− 0.02	− 10	− 6.6	− 8.6	− 7.3	− 8.6
Calcite	CaCO_3_	*	− 1.0	− 0.99	***− 0.34***	− 0.71	2.1	− 2.0	***0.43***	− 0.67	***− 0.22***	***− 0.30***
CaMoO_4_		− 1.8	− 3.2	***− 0.38***	− 1.3	− 1.3	− 1.2	− 3.1	− 0.75	− 2.6	− 3.2	− 2.3
FeMoO_4_		***− 0.11***	− 5.1	0.91	***− 0.07***	− 1.00	− 15	− 2.7	− 3.1	− 2.2	− 2.0	− 2.9
Celestite	SrSO_4_	− 1.6	− 1.1	− 1.5	− 1.6	− 0.99	− 0.99	− 2.3	− 1.1	− 2.4	− 1.3	− 1.9
Diaspore	Al(OH)_3_	***0.45***	1.5	4.6	2.6	1.7	− 1.4	1.2	1.5	1.3	1.3	1.9
Fe(VO_3_)_2_		− 11	− 8.7	− 2.4	1.0	− 5.5	− 22	− 7.4	− 3.0	*	− 5.0	− 3.4
Gibbsite	Al(OH)_3_	− 0.93	***0.15***	3.2	1.2	***0.32***	− 2.8	***− 0.11***	***0.15***	***− 0.07***	***− 0.08***	0.57
H-Jarosite	(H_3_O)Fe_3_(SO_4_)_2_(OH)_6_	− 9.3	− 6.2	1.2	3.8	− 5.1	− 25	− 6.7	− 8.4	− 3.9	***0.46***	− 7.2
K-Jarosite	KFe_3_(SO_4_)_2_(OH)_6_	− 4.2	0.77	8.3	11	1.9	− 14	***− 0.42***	***− 0.34***	2.0	7.6	− 0.62
Siderite	FeCO_3_	*	− 3.2	***− 0.08***	***0.47***	− 0.74	− 12	− 1.9	− 2.3	− 0.61	0.57	− 1.3
Uraninite	UO_2_	− 0.95	− 8.6	− 4.7	− 5.6	− 5.7	− 15	− 6.6	− 5.7	− 5.7	− 8.2	− 6.3

SI in the range − 0.5 to + 0.5 are given in bold italics

^*^Ligand levels below limit of detection

#### Other high concentration elements—Al, Fe, Mn, Li and Sr

Highest concentrations of iron are found in the acidic G1 (reaching 379 mg/L in 2015–2019), and G3 (167 mg/L) and G10 (202 mg/L) have high concentrations (Table 6) indicating the presence of ferrous iron. In the case of G10, the pipe is made of steel, but presumably, the high concentrations are explained to a large part by water affected by alum shale. The ditch north of the waste deposit shows spots with both elevated temperatures and electrical conductivity, which indicates that leachates from warm parts of the deposit are reaching the ditch through the groundwater. Iron concentrations in the ditch are much lower though, than in the groundwater, supposedly due to precipitation of iron oxyhydroxides when reaching neutral and oxidizing surface water (cf. Armstrong et al. 2019).

A comparison between all sampling occasions in all groundwaters indicates a negative correlation with pH for manganese (*r*^2^ 0.78, logged data) and a weaker negative correlation (*r*^2^ 0.53) for iron (logged data). Other elements show even weaker correlations with pH. Perkins and Mason (2015) found that manganese is highly mobile in weathering shale and suggested that manganese loss from carbonate poor black shales can be an indicator of weathering even if no other signs of weathering are clearly noted. In Kvarntorp, G2, G7 (the deep wells) and G6 (highest pH) have manganese concentrations in the range 0.0002–0.4 mg/L; G9 (local background reference) generally has below 1 mg/L and G8 normally around 1 mg/L. Groundwaters G5, G10 and G11 have concentrations between 1 and 6 mg/L while G1 and G3 have concentrations between 10 and 22 mg/L. Groundwaters with elevated manganese concentrations in Kvarntorp could be due to weathering and/or reducing conditions in the groundwater.

Lithium concentrations of about 5 μg/L (median) in the reference background well G9 are below the levels in the sandstone aquifer (10–60 μg/L in G2 and G7). Groundwaters G5, G6, G8, G10 and G11 have concentrations between 10 and 600 μg/L, while G1, G3 and G4, that are believed to be highly affected by the shale leachate, have concentrations between 1000 and 2000 μg/L. Lithium concentrations in alum shale has previously been reported to be in the range 4- to 24-mg/kg dw (Allard et al. 2011), which means that alum shale is not enriched in lithium compared to other black shales (world median 31-mg/kg dw; Ketris and Yudovich 2009). Leaching tests have shown that lithium is quite easily leached from alum shale, but not from shale ash (Åhlgren et al. in preparation), which strengthens the suggestion of contact with partly pyrolysed shale or fines for G1 and G3 but possibly also for G4. Lithium has been reported to be found in high concentrations in flowback water after fracking in the Marcellus shale (USA) with median concentrations of 95 mg/L after 14 days (Haluszczak et al. 2013) and is considered for recovery (Lee and Chung 2020). These concentrations largely surpass the groundwater in Kvarntorp.

Regional strontium background level in surface water upstream the Kvarntorp area is below 0.1 mg/L (Åhlgren et al. 2020). Only the upstream well G9 has concentrations that low. Groundwaters G1, G3, G4, G7, G10 and G11 have concentrations between 0.2 and 0.7 mg/L, while G5, G6 and G8 exceed 1 mg/L. Highest concentrations are found in the deep groundwater G2 (1.5–2.2 mg/L). Even though the strontium concentrations are higher in the deep groundwater, the levels are not high when comparing with Danish drinking water works (median 1.45 mg/L, range 0.02 to 30 mg/L) and Danish groundwater wells (median 1.52 mg/L) (Frei et al. 2020).

#### Hydrolyzable divalent trace elements—Co, Ni, Cu and Zn

**Fig. 7:**
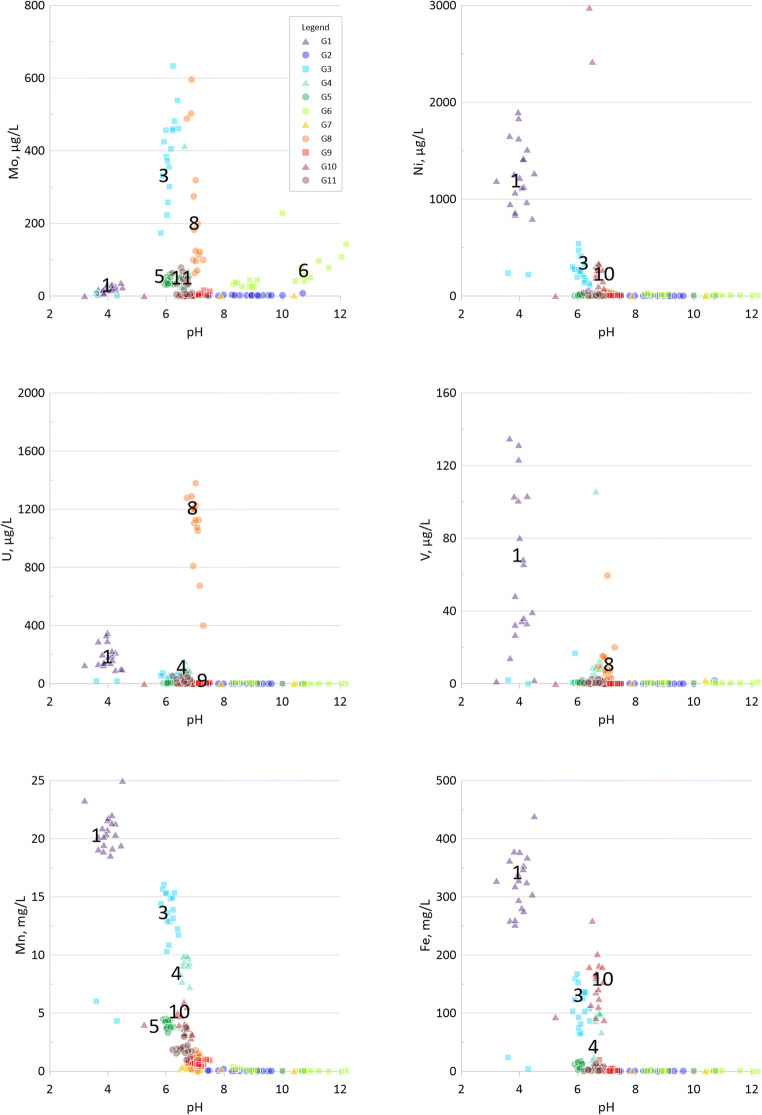
Diagrams showing concentrations vs. pH for hydrolysable major elements and trace elements (Mn, Fe and Ni) as well as redox sensitive trace elements (V, Mo and U) in groundwaters G1–G11

Concentrations of nickel (as well as manganese, iron, vanadium, molybdenum and uranium) as a function of pH are given in Fig. 7. A pH influence is evident for nickel, as expected, with high concentrations at low pH (G1) decreasing with increasing pH (G3, G4 and G10). The higher levels in G10 in comparison with G3 and G4 may indicate reducing conditions.

Total concentrations of the hydrolysable elements cobalt, nickel, copper and zinc are high in fines and shale ash (Table 5). Concentrations of cobalt, nickel and zinc in the shale waters from G1 to G3, with low pH, are higher than in the other groundwaters (Table 7), even though for nickel, the correlation with pH is negative but fairly weak (*r*^2^ 0.32) when all groundwater wells are included. High levels of cobalt and nickel are observed in G10. High concentration of particularly nickel, but also cobalt and zinc, in the groundwaters are evidently indications of shale weathering and contribution of contaminated leachates. Soils underlain by black shales elsewhere have shown to be enriched in easily mobilized metals with cadmium and copper being of particular concern (Yu et al. 2012), and cadmium has been found in elevated concentrations in rice in black shale areas (Duan et al. 2020). Both cadmium (median level 12 μg/L) and copper (median level 460 μg/L) have been found at high concentrations in Degerhamn in acidic groundwater (Lavergren 2008), whereas in Kvarntorp, the median for cadmium is lower (6 μg/L for G1) and copper much lower (median 2.4 μg/L for G1 and 5.5 μg/L for G3). Part of the explanation could be in concentration differences in the shale material, since both elements display higher concentrations in the Degerhamn shale bedrock (3-mg/kg dw cadmium and 170-mg/kg dw copper; Lavergren 2008) compared to Kvarntorp (median 1.14-mg/kg dw cadmium and 137-mg/kg dw copper in drill cores).

Leaching tests (Åhlgren et al. 2018) with fines and shale ash (the same material as given in Table 5) showed that nickel was not released from the shale ash to any greater extent, whereas there was a significant release of nickel from the fines at low pH.

#### Redox and pH sensitive trace elements—V, As, Mo and U

Kvarntorp alum shale is enriched in the redox sensitive elements vanadium, arsenic, molybdenum and uranium (Table 5). Elevated concentrations of these elements in the groundwaters (Table 7) are indicators of shale weathering and further mobilization into the groundwater (see Fig. 4 for the spatial distribution).

Vanadium and arsenic can be incorporated in pyrite which is the probable origin of these elements in alum shale. In addition, vanadium is also trapped in clays (Lerat et al. 2018). Shale residues from the waste deposit show a range of 420- to 750-mg/kg dw vanadium and 80- to 150-mg/kg dw arsenic, and these elements have a correlation of 0.49 (*r*^2^, logged data) in the groundwaters. It is, however, not the same groundwater well that has the highest concentrations of both vanadium and arsenic. Neither of these two elements seems to be leached to any greater extent, except for vanadium in G1 with up to 130 μg/L and arsenic in G3 with up to 170 μg/L. This is in line with results from studies of alum shale, shale residues and groundwater in Degerhamn, Öland, which also showed low leachability of arsenic and vanadium (Lavergren 2008). Leaching tests at pH 12, however, have shown more important leaching of vanadium from shale ash where 28% of the vanadium was leached (Karlsson et al. 2013) confirming that vanadium is most mobile under oxidized and alkaline conditions (Wright and Belitz 2010). Arsenic mobility in fractured rock with pyritic black shales has earlier shown to be determined by concentrations of sulfide and available oxidant where microbial sulfide production is suggested to enhance arsenic mobilization (Zhu et al. 2008).

Yu et al. (2014) suggest that arsenic released from oxidation of arsenian pyrite is adsorbed onto the surface of, or coprecipitated with, secondary iron minerals. A possible explanation for the differences in arsenic concentrations in G1 and G3 could be precipitation of schwertmannite in G1 favoured by the lower pH. Arsenic is effectively adsorbed to schwertmannite (Acero et al. 2006), and arsenic is thus removed from the groundwater through either coprecipitation or sorption to schwertmannite in G1. In 2004, arsenic concentrations and pH were lower in G3, indicating possible precipitation of schwertmannite and removal of arsenic also in G3 at that time.

Molybdenum has reduced mobility in sulfidic environments due to the formation of MoS_2_ simultaneously with FeS and FeS_2_ (Smedley et al. 2014). Molybdenum, acting as the molybdate anion with concentrations of 130- to 210-mg/kg dw in fines and ashes (Table 5) shows restricted solubility at low pH and has increased from below 5 to 170–630 μg/L in G3 since the pH increased from 3.6–4.3 in 2004 to 5.8–6.4 in 2015–2019. Molybdenum concentrations are below 36 μg/L, despite the presumed high weathering rate in the well, which could be due to the low pH. G6, with median pH above 9, has fairly high concentration of molybdenum (maximum level above 100 μg/L), which would be due to the high pH, while G8 shows even higher concentrations (maximum level almost 600 μg/L for the period 2015–2019).

Molybdenum concentrations in G6 show enhanced leaching at high pH, whereas in G3 and G8, redox processes are the likely reason for elevated concentrations. Uranium displays leaching due to low pH in G1 and governed by high carbonate levels in G8 where it covaries with molybdenum but not iron. Also vanadium is leached due to low pH in G1.

It is interesting to note that molybdate might be solubility controlled by for instance CaMoO_4_ and FeMoO_4_. (Table 8). There is no apparent solubility control of strontium by celestite (SrSO_4_), nickel by NiMoO_4_, vanadium by Fe(VO_3_)_2_ or uranium by uraninite (UO_2_).

Uranium occurs in four manners in alum shale according to Armands (1972): (1) in authigenic K-feldspar, (2) in detrital zircon and titanite, (3) with sulfur and pyrite and (4) organically bound to carbon, whereas Lecomte et al. (2017) propose that uranium was accumulated in organic matter and biogenic phosphate nodules. Uranium concentrations in shale ash and fines are in the range 160- to 240-mg/kg dw. Elevated concentrations of uranium are found in G8 (up to 1760 μg/L) which has a circumneutral pH but the highest carbonate concentrations of all waters, indicating formation of soluble uranium carbonate complexes (Bernhard et al. 1996). High uranium concentrations have previously been observed in one of the pit lakes in the Kvarntorp area, with circumneutral pH and high carbonate level (Allard et al. 2011; Åhlgren et al. 2020).

Leaching tests (Åhlgren et al. 2018) with fines and shale ash (the same material as given in Table 5) showed that uranium leached from both fines and ashes at low pH.

### Temperatures

Temperatures were elevated in groundwaters at the north-eastern part of the deposit, both in 2005 and in 2017. Fig. 4, bottom right, shows the temperatures at a depth of 1 m below the water surface in November 2017. Measurements in the ditch just north of the deposit show higher temperatures (18 °C in June 2019) close to well G3 than further to the west (12 °C), indicating that warm water from the deposit is reaching the ditch at this site. Temperatures in the groundwater have not changed notably since 2005. Either the heat generating reactions in the waste deposit have not decreased, or the heat storage capacity in the waste deposit is so significant that the cooling process is very slow. Modelling of the temperature decrease performed in 2005 (Holm et al. 2005) indicated an expected cooling time period of up to several hundred years when treating the waste deposit as a passive heat storage. This estimate most likely underestimated the time period as the presence of reactive shale with chemical energy has the capacity to produce energy as heat was not included in this modelling.

### Time trends

Generally, there is no evident time trend indicating significant changes in releases of metals from the shale to groundwaters G1–G6, G8, G10 and G11. The greatest disparity between different years is found when data from 2004 are compared with those from 2015–2019, as, for example, much higher cobalt and nickel concentrations in G10 in 2004 compared to 2015–2019. When comparing results only from 2015–2019 vanadium, nickel and uranium display a decreasing trend in G1. When results for 2004 are included, the trend is not evident since some of the concentrations from that year are lower than in 2015. G8 displays varying concentrations for molybdenum. A possible increased weathering during 2015–2019 compared to 2004 is suggested for G3 and G4 (both downstream the deposit) since both electrical conductivity and Mg/Ca ratios agree with such a conclusion.

G10 shows a decrease in sulfate (from 3000 to around 2000 mg/L), magnesium (from 400 to around 150 mg/L), cobalt (from > 300 to 1–67 μg/L) and nickel (from > 2400 to 4–300 μg/L) after 2004. This may be an indication that this well is presently less affected by shale weathering. The well is situated upstream the deposit and is not expected to receive leachates from the deposit itself but rather from the surrounding dumped fines through surface run-off.

The deposit was still unvegetated until the beginning of the 1980s, but vegetation has gradually colonized the deposit since then, and today, primarily birches (*Betula*) are common. Other sites have shown dissolution of goethite and release of trace elements due to reductive conditions caused by organic cover (Paktunc 2013) and leaching tests of alum shale fines from Kvarntorp have shown enhanced leaching of vanadium and molybdenum when covered by organic material (Sjöberg and Karlsson 2015).

A general conclusion is that changes in groundwater chemistry with time in terms of increasing or decreasing levels of contaminants originating from the deposit cannot be quantitatively assessed. There are at least three uncertainties that prevent predictions of the development with time: (1) lack of data from the initial period from 1942 to 2003. (2) Uncertainties in the prediction of how the temperature in the deposit will develop with time. Wet or even water saturated parts of the deposit will be depleted with respect to leachable elements, but significant parts of the deposit are still hot and dry. An open question remains whether releases of elements will increase or possibly decrease when the deposit cools down and more water starts to infiltrate. (3) The heterogeneity of the deposit with three categories of shale residues with different weathering potential. The exact locations and distribution of these different categories within the deposit are not known in detail. However, it is reasonable to expect effects on the releases of element-enriched leachates from the deposit area once it has cooled down and is infiltrated with water in all parts.

## Conclusions

The shallow groundwaters around the Kvarntorp waste deposit (upper aquifer, in the till horizon) show clear impacts from shale waste in terms of elevated levels of elements which obviously are released from weathering shale and shale ashes. PCA of the groundwater data in combination with leaching data from humidity cells of specific waste materials from the area provides information about potential sources for trace elements to the groundwater. This analysis confirms assessments solely based on water monitoring data combined with data regarding the water sampling wells (locations, depths, lining etc). For eight of the sampled groundwaters, the concentrations of elements characteristic of alum shale, such as molybdenum, nickel, uranium and to some extent also arsenic, are well above background levels by up to three orders of magnitude (vanadium in one well with pH below 4). Groundwater in the lower aquifer (depth 31 m, in the sandstone layer) is generally not affected.

Presence of groundwater with low pH (below 4) in particularly one groundwater indicates high loadings of leachates from fines containing pyrite that is being oxidized, which produces acid. Presence of highly alkaline pH (up to 12) in particularly one groundwater is suggested to be due to presence of calcium hydroxide from the degradation of calcium carbonate at high temperatures (above 500 °C) in the deposit. Most of the waste is composed of shale ash which also left signatures in the water like arsenic, molybdenum, uranium and to some extent vanadium, however, at near neutral pH (above 6.5).

Concentrations in the shallow groundwaters downstream the deposit reached high levels, e.g., up to 172-μg/L As, 633-μg/L Mo, 1900-μg/L Ni, 1760-μg/L U, 135-μg/L V and 4400-mg/L SO_4_^2−^. Substantial quantities of these elements are released from the waste deposit. If distributed into the regional groundwater system, such concentrations could constitute a potential health hazard, but the groundwater distribution seems to be rather limited since the lower aquifer is not affected.

Evidently, there is a continued and progressing impact of shale leachates on the groundwater around the deposit since at least 2004 and probably during the whole lifetime of the deposit since the 1940s. The time span between 2015 and 2019 is too short to distinguish a significant trend. There are indications of increased activity and weathering in some of the wells, supported by both increased electrical conductivity and increased Mg/Ca ratios in combination with increased potassium concentrations. Most groundwaters, though, do not show any clear changes in levels of elements from shale waste weathering which suggests that no general leaching trend for the area can be discerned based on data from the short time span 2004–2019. A reasonable conclusion is, nevertheless, that leaching will progress at about the same level for a long time and that the current release of elements to downstream water will continue. The waste deposit has affected the surroundings for more than half a century and will probably continue to do so, possibly for centuries to come.

Presence of different waste types makes the picture in Kvarntorp complex, but it has been demonstrated that processed black shale has long-term impact on the surrounding environment with high levels of several elements, notably arsenic, molybdenum, uranium and to some extent nickel.

## Data Availability

The datasets used and/or analysed during the current study are available from the corresponding author on reasonable request.
